# Supplementary material for: Efficient 3D printing via photooxidation of ketocoumarin based photopolymerization
Source: Nat Commun. 2021 May 17;12:2873. doi: 10.1038/s41467-021-23170-4 (PMC8129151; doi:10.1038/s41467-021-23170-4)
Supplement: Supplementary file 1 — Supplementary Information [file 41467_2021_23170_MOESM1_ESM.pdf]

SUPPLEMENTARY INFORMATION

Efficient 3D printing via photooxidation of ketocoumarin based photopolymerization

Xiaoyu Zhao,[1,2] Ye Zhao,[1,2] Ming-De Li,[3] Zhong'an Li,[1] Haiyan Peng,[1,2,*] Tao Xie,[4] Xiaolin Xie[1,2,*]

[1]Key Lab for Material Chemistry of Energy Conversion and Storage, Ministry of Education, School of Chemistry and Chemical Engineering, Huazhong University of Science and Technology (HUST), Wuhan 430074, China

[2]National Anti-Counterfeit Engineering Research Center, HUST, Wuhan 430074, China

[3]Key Laboratory for Preparation and Application of Ordered Structural Materials of Guangdong Province, Department of Chemistry, Shantou University (STU), Shantou 515063, China

[4]State Key Laboratory of Chemical Engineering, College of Chemical and Biological Engineering, Zhejiang University (ZJU), Hangzhou 310027, China

[*]Corresponding authors: hypeng@hust.edu.cn (H. Y. P.), xlxie@hust.edu.cn (X. L. X.)

1. Supplementary Notes

Supplementary Note 1 | Lateral photopolymerization

Because of the expanded light path of digital light and light scattering by microparticulate solids formed during printing, lateral photopolymerization usually occurs in the area beyond that predesigned (see Supplementary Fig. 32), enlarging the solidified areas and decreasing the print resolution.[1] Therefore, it is valuable to decrease the light transmittance of solidified polymer parts and the light penetration depth so that the lateral photopolymerization can be depressed and the print resolution can be increased. Within the visible light wavelength region, the minimum light transmittance is 62% and 42% for the polymer films (thickness: 20 μm) photomediated by KCD/NPG and KCD/TA, respectively, indicating a decreased light penetration and weakened lateral photopolymerization for the latter. Consequently, the feature size of the square lattice is barely identifiable when photomediated by the KCD/NPG system, while the KCD/TA system generates a much higher print resolution.

Supplementary Note 2 | Calculating the wasted light energy by nonreactive light absorbers during 3D printing

Absorbance (Abs) of the nonreactive light absorbers (NLA) and photoinitiators (PI) in the printing resin can be calculated according to the Beer-Lambert law:

$$\text{Abs} = \varepsilon_{\text{NLA}} D[\text{NLA}] + \varepsilon_{\text{PI}} D[\text{PI}] = -\log(\frac{E}{E_0}) \tag{1}$$

where, ε_{NLA}, ε_{PI}, [NLA] and [PI] are the molar excitation coefficients and concentrations of NLA and PI, respectively; D is the penetration depth; E_0 and E are the incident and transmitted light energies, respectively. Then, the absorbed light energy (E'_{a}) by the NLA and PI can be calculated as follows:

$$E'_{\text{a}} = E_0 - E = E_0 - E_0\exp(-2.3\varepsilon_{\text{NLA}} D[\text{NLA}] - 2.3\varepsilon_{\text{PI}} D[\text{PI}]) \tag{2}$$

Thus, the differential of E'_{a} by the penetration depth D can be expressed as:

$$E_{\text{a}} = \frac{\text{d}E'_{\text{a}}}{\text{d}D} = E_0(2.3\varepsilon_{\text{NLA}}[\text{NLA}] + 2.3\varepsilon_{\text{PI}}[\text{PI}])\exp(-2.3\varepsilon_{\text{NLA}} D[\text{NLA}] - 2.3\varepsilon_{\text{PI}} D[\text{PI}]) \tag{3}$$

With respect to a very thin layer of the printing resin (i.e., D is very small), the absorbed light energy can be calculated from a simplified equation:

$$E_{\text{a}} = E_0(2.3\varepsilon_{\text{NLA}}[\text{NLA}] + 2.3\varepsilon_{\text{PI}}[\text{PI}]) \tag{4}$$

Therefore, the light absorption efficiency (φ_{NLA}) of NLA can be calculated from the following equation:

$$\varphi_{\text{NLA}} = \frac{E_{\text{NLA}}}{E_{\text{a}}} = \frac{\varepsilon_{\text{NLA}}[\text{NLA}]}{\varepsilon_{\text{NLA}}[\text{NLA}] + \varepsilon_{\text{PI}}[\text{PI}]} \tag{5}$$

The calculated value of φ_{NLA} is based on the reported parameters of NLA and PI by Harper and Chmely and co-workers (Supplementary Table 1).[2]

Supplementary Note 3 | Calculating the light energy efficiency of 3D printing

The light energy efficiency (φ) of 3D printing can be calculated as follows:

$$\varphi = \psi_{\mathrm{PI}}\varphi_{\mathrm{PI}} = \psi_{\mathrm{PI}}(1 - \varphi_{\mathrm{NLA}}) \tag{6}$$

where, ψ_{PI} is the photoinitiation efficiency, φ_{PI} is light absorption efficiency of the photoinitiation system. ψ_{PI} is reported to be 0.40 for diphenyl(2,4,6-trimethylbenzoyl)phosphine oxide that is widely used in the DLP 3D printing as a type I photoinitiator.[3] This number is generally larger than that of type II photoinitiators which show lower reactivity.[4,5] Thus, with respect to the printing resin containing the NLA, the light energy efficiency of 3D printing is believed to be ~ 2% based on the data shown in Supplementary Table 1.

For the KCD/NPG system, ψ_{PI} is estimated to be 0.2.[6,7] Since no NLA was added, the light energy efficiency φ of 3D printing should be 20%.

ψ_{PI} of the KCD/TA system can be calculated using the following equation:[8]

$$R_{\mathrm{p}} = k_{\mathrm{p}}(1 - \alpha)\left(\frac{2.3\varepsilon_{\mathrm{PI}}[\mathrm{PI}]I_0\psi_{\mathrm{PI}}}{k_{\mathrm{t}}}\frac{\lambda}{N_{\mathrm{A}}hc}\right)^{0.5} \tag{7}$$

where, R_{p} is the polymerization rate; k_{p} and k_{t} are the rate constants of propagation and termination, respectively; I_0 and α are the incident light intensity and double-bond conversion, respectively; λ, N_{A}, h and c are the light wavelength, Avogadro's number, Planck's constant and light speed, respectively. ψ_{PI} is estimated to be 0.24 for the KCD/TA system, by comparing the R_{p} with that of the KCD/NPG system using the same resin. To do this, the KCD concentration is set to be identical in both systems, and the concentration of TA and NPG is the same. In addition, a small (e.g., 5%) double bond conversion is used for the calculation where the inhibition effect of the ketyl radical can be negligible (Supplementary Fig. 33). Therefore, the light energy efficiency φ of 3D printing for the KCD/TA system is estimated to be 24%.

Supplementary Note 4 | Nanosecond transient absorption spectroscopy.

Nanosecond transient absorptions were characterized in the flow mode and toluene was used as the solvent. During characterization, an integrated femtosecond laser amplifier system comprising amplifier, femtosecond oscillator and pump laser was used, which performed under the temperature of 20 °C. The laser has a lifetime of about ten thousand hours and a horizontal polarization with a polarization ratio of >100:1. The beam spatial profile is shown in Supplementary Fig. 34. The resolution of the spectrometer is 0.12 ps. During the photooxidation of KCD by TA, a 400 nm pump laser was employed to trigger the photoreaction so that only KCD could be excited. To increase the signal intensity without significantly sacrificing the accuracy, the content of KCD and TA was optimized to be 175 μM and 3.5 mM, respectively.

The lifetime of triplet KCD is expected to decrease when increasing its concentration due to the increased reaction possibility. To verify this assumption, we recorded the transient absorption upon laser irradiation with consecutively varied power (e.g., 0.3, 0.6, 1.2, 1.8 and 2.4 mW). Transient absorptions at 600 nm were used to calculate the lifetime due to that the background could be eliminated.

To obtain the nanosecond transient absorption of pure TA (175 μM) in the absence of KCD, a 330 nm pump laser was employed to excite the TA molecules and to provide a significant signal. Continuous argon gas was purged into the solution to remove oxygen during all transient absorption experiments.

Supplementary Note 5 | Rheology.

Viscosity of the printing resin was measured on a rotational rheometer (MCR 302, Anto-Parr, Austria). The sample thickness and chamber temperature were 0.1 mm and 298 K, respectively. The sheer rate was increased from 10 s^{-1} to 1000 s^{-1} during measurement.

Supplementary Note 6 | Tensile test.

Tensile test was conducted on an MTS testing machine (Exceed Model E44, United States). Samples in the Type IV dumbbell shape were prepared to evaluate their mechanical properties (GB/T 528-2009/ISO 37:2005). The strain rate was set to be 1 $mm \cdot min^{-1}$.

Supplementary Note 7 | Computation.

Density functional theory (DFT) calculations were performed using the Gaussian 09 Software.[9] All chemical structures were simplified to save the computation resources by replacing alkyl groups with the methyl group. For details, the structures of KCD, IM1, IM2, IM3 and IM4 (Supplementary Fig. 3) were simplified as M_KCD, M_IM1, M_IM2, M_IM3 and M_IM4, respectively.

To calculate the absorption spectra of M_KCD S_1, M_KCD T_1, M_IM1, M_IM2, M_IM3, M_IM4 and $TA^{\bullet}$, the M06-2X hybrid function was used. The chemical structures in both ground and excited states were optimized using the 6-311G(d,p) basis set. Analytic frequency calculations were employed, and no imaginary frequency was found, indicating that the stationary point was reached. The absorption spectra were calculated using the 6-311+G(d,p) basis set by time-dependent density functional theory (TD-DFT) with the polarizable continuum model (PCM, toluene). The calculated spectra were all scaled by 1.23 times.

To calculate the oxidation potential of M_IM2 and M_IM3, a method was employed as previously reported by Guo and co-workers.[10] In detail, the Supplementary Equation 8 and B3LYP hybrid function were used during calculation. The structures were optimized with the 6-31+G(d) basis set. Analytic frequency calculations were also conducted to confirm the stationary point. In addition, the single point energy in gas-phase was calculated with the 6-311++G(2df,2p) basis set. The solvation free energy was calculated using the 6-31+G(d,p) basis set with the SMD model (solvent: AcCN, alpha: 1.2, radii: bondi).[11]

$$E^0(vs\,\mathrm{NHE}) = (\mathrm{IP_{B3LYP}} + \mathrm{IP_{corre}}) + \frac{1}{A}\left(-T\Delta S + \Delta G_{\mathrm{solvation\_ox}} - \Delta G_{\mathrm{solvation\_red}}\right) - \Delta G_{\mathrm{NHE}} \qquad (8)$$

where, $\mathrm{IP_{B3LYP}}$ was the gas-phase adiabatic ionization potential calculated by the B3LYP hybrid function (unit: eV), T was the temperature (unit: K), ΔS was the entropy variation when changing

from the reduced form to the oxidized form in the gas-phase at 298 K (unit: kcal·mol^{-1}). $\Delta G_{\mathrm{solvation\_ox}}$ and $\Delta G_{\mathrm{solvation\_red}}$ represented the solvation free energy (unit: kcal·mol^{-1}) of the oxidized and reduced forms, respectively. $\mathrm{IP}_{\mathrm{corre}}$ and ΔG_{NHE} were the correlation value (0.28 eV)[10] for $\mathrm{IP}_{\mathrm{B3LYP}}$ and the free energy change (4.44 eV)[12] associated to the half reaction of the normal hydrogen electrode, respectively. A was the conversion coefficient (23.06) between kcal·mol^{-1} and eV.

Supplementary Note 8 | Proposed mechanism for the photoreaction of KCD with TA

No byproducts associated with oxygen were detected due to that oxygen was removed by continuous argon gas purge. As illustrated in Supplementary Fig. 3, upon visible light irradiation, KCD is excited into its first singlet excited state (S_1) and then quickly transforms into its first triplet state (T_1) *via* ISC. Subsequently, the triplet KCD donates one electron to TA. Given that TA has a high reduction potential (-1.00 V *vs* SCE),[13] it readily accepts the electron from the triplet KCD. After electron transfer, the triplet KCD converts to a cationic radical intermediate (IM1), while the carbon-chloride bond of TA is broken through heterolytic cleavage to release one radical ($TA^{\cdot}$) and one Cl^-.[14] Then the produced Cl^- grabs one proton from the amino-alkyl group of IM1, yielding a radical intermediate (IM2).[14,15] Electron transfer from IM2 to TA is thermodynamically unfavorable due to that the oxidation potential of IM2 (-0.31 V *vs* SCE, according to the DFT calculation) is much higher than the reduction potential of TA (-1.00 V *vs* SCE). Feasibly, IM2 is ready to be oxidized by the triplet KCD (ΔG_{ET} = -1.28 eV), based on the reduction potential (-1.23 V *vs* SCE)[16] and triplet energy (2.20 eV)[17] of KCD. Consequently, an anionic radical intermediate (IM3) and a cationic intermediate (IM4) are simultaneously produced. IM3 exhibits a low oxidation potential (-1.46 V *vs* SCE according to DFT calculations), so that it readily denotes one electron to TA, forming the stable KCD and another molecule of the radical $TA^{\cdot}$. At the same time, IM4 can readily react with water (remained in the solvent and monomer, Supplementary Table 2), yielding the final product KCD_2 and releasing one molecule of acetaldehyde (CH_3CHO).

To further verify the proposed mechanism, we conducted nanosecond transient absorption spectroscopy upon a 400 nm pump laser irradiation. Only KCD was supposed to be excited under such a condition, because TA exhibits no absorption around 400 nm (Supplementary Fig. 21). To

evaluate the potential influence of the radical TA˙ on the transient absorption upon excitation by 400 nm light, we measured the transient absorption of pure TA upon 330 nm laser irradiation. Results show that the radical TA˙ can only last for 7.6 ns in toluene (Supplementary Fig. 5), showing a negligible influence on the transient absorption of the KCD/TA system. This is mainly because that the radical TA˙ is readily quenched by toluene, which is consistent with the results of the HPLC-HRMS characterization (Supplementary Fig. 9).

As shown in Supplementary Fig. 4a, upon the 400 nm pump laser irradiation, KCD shows a positive absorption peaked at 490 nm, along with negative absorptions below 470 nm. The negative absorption was caused by the ground-state bleach of KCD.[16,18] The positive absorption peak is assigned to the singlet KCD (S_1), as supported by the DFT calculation (Supplementary Fig. 4g). Subsequently, peak absorptions bands at 515 and 587 nm, and the broad absorption band above 660 nm gradually appear at ~1.16 ns (Supplementary Fig. 4b), indicating the generation of the triplet KCD (T_1) through ISC, this assignment is also supported by the result of DFT calculation (Supplementary Fig. 4g) and earlier reports.[16,18] Subsequently, a characteristic absorption band peaked at 495 nm appears at ~487 ns (Supplementary Fig. 4c), indicating the electron transfer from the triplet KCD to TA and the production of IM1 (Supplementary Fig. 4g). After that, the 495 nm peak absorption red shifts to 500 nm at ~1.71 μs (Supplementary Fig. 4d), indicating the generation of IM2 through the proton abstraction by Cl^- from IM1 (Supplementary Fig. 4g). At the same time, the absorption band peaked at 523 and 575 nm appear, suggesting the formation of IM4 and IM3, respectively (Supplementary Fig. 4g), through the electron transfer from IM2 to triplet KCD. The peak at 575 nm is not significant as expected due to the low content of IM3, which is probably due to the rapid electron transfer from IM3 (E_{ox}, -1.46 V *vs* SCE according to DFT calculations) to TA (E_{red}, -1.00 V

vs SCE). Notably, the concentration of triplet KCD is increased when increasing the pump laser power, which is supported by the increased ΔOD (Supplementary Table 5). However, the lifetime of triplet KCD is gradually decreased, suggesting a promoted reaction of the triplet KCD with IM2. With time going on, the absorption peaks centered at 500, 575 and 730 nm decay toward the baseline, but the 523 nm peak associated with the IM4 remains at ~8.82 μs (Supplementary Fig. 4e). Finally, the absorption band of IM4 at 523 nm disappears at ~42.7 μs due to the reaction with H_2O (Supplementary Fig. 4f).

2. Supplementary Tables

Supplementary Table 1 | Roughly estimated light energy ratio (φ_{NLA}) consumed by NLA[a]

PI		NLA		φ_{NLA} (%)	Reference
Content (mM)	ε ($L \cdot mol^{-1} \cdot cm^{-1}$)	Content (mM)	ε ($L \cdot mol^{-1} \cdot cm^{-1}$)		
11.48	6.0×10^2 (380 nm)	3.72	5.0×10^4 (372 nm)	96	[2]

[a] Peak absorptions were used for the calculation.

Supplementary Table 2 | Water content in toluene, DMAA, TMPEOTA and PETTA

Substrate	Toluene	DMAA	TMPEOTA	PETTA
H_2O content (mM)	96	170	78	67

Supplementary Table 3 | Tensile strength, tensile modulus and elongation at break of 3D printed samples before and after postcuring

Entry	Before postcuring			After postcuring		
	Tensile strength (MPa)	Tensile modulus (MPa)	Elongation at break (%)	Tensile strength (MPa)	Tensile modulus (MPa)	Elongation at break (%)
1	19	110	38	44	276	27
2	24	96	34	42	232	27
3	20	124	38	35	265	29
4	22	118	40	39	268	29
5	21	114	32	39	259	34
Average	21±2	112±10	36±3	40±3	260±15	29±2

Supplementary Table 4 | Reported print speed and print resolution

No.	Print speed ($cm\cdot h^{-1}$)	Print resolution (μm)	DLP printer	Reference
1	2.5	50	Continuous 3D printer	[19]
2	43.2	300	Continuous 3D printer	[1]
3	-	10	Equipped with LCoS and lens	[20]
4	32.7	100	Equipped with lens	[21]
5	2.4	18	Common DLP 3D printer	[22]
6	1.8	100	Common DLP 3D printer	[23]
7	1.2	100	Common DLP 3D printer	[24]
8	0.05	-	Common DLP 3D printer	[25]
9	0.13	-	Common DLP 3D printer	[26]
10	1.2	-	Common DLP 3D printer	[27]
11	9.1	200	Common DLP 3D printer	[28]
12	**5.1**	**23**	Common DLP 3D printer	**This work**

Supplementary Table 5 | Maximum ΔOD and lifetime of triplet KCD upon irradiation with varied pump laser power

Pump laser power (mW)	0.3	0.6	1.2	1.8	2.4
Maximum ΔOD (10^{-3})	2.29	4.25	7.79	13.70	17.70
Lifetime (μs)	0.87	0.80	0.76	0.59	0.55

3. Supplementary Figures

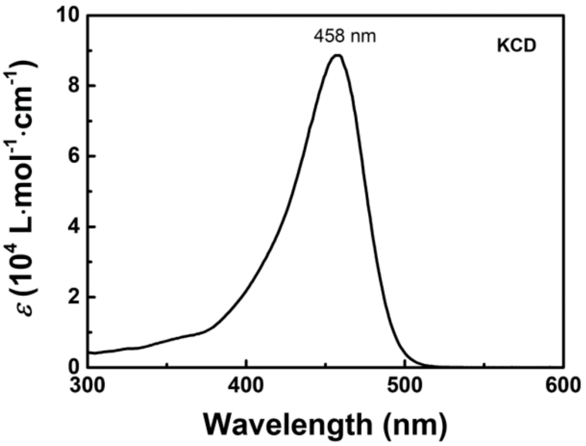

Supplementary Figure 1 | UV-vis absorption spectrum of KCD.

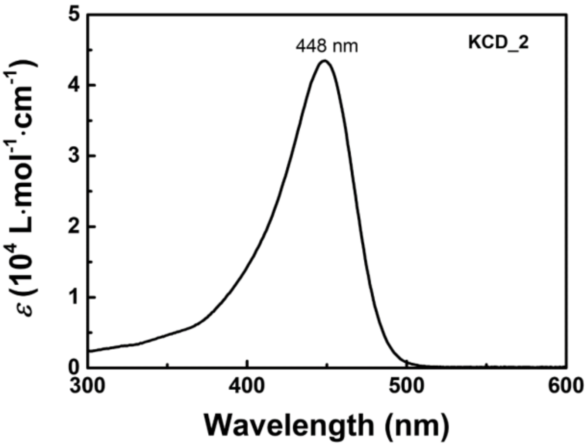

Supplementary Figure 2 | UV-vis absorption spectrum of KCD_2.

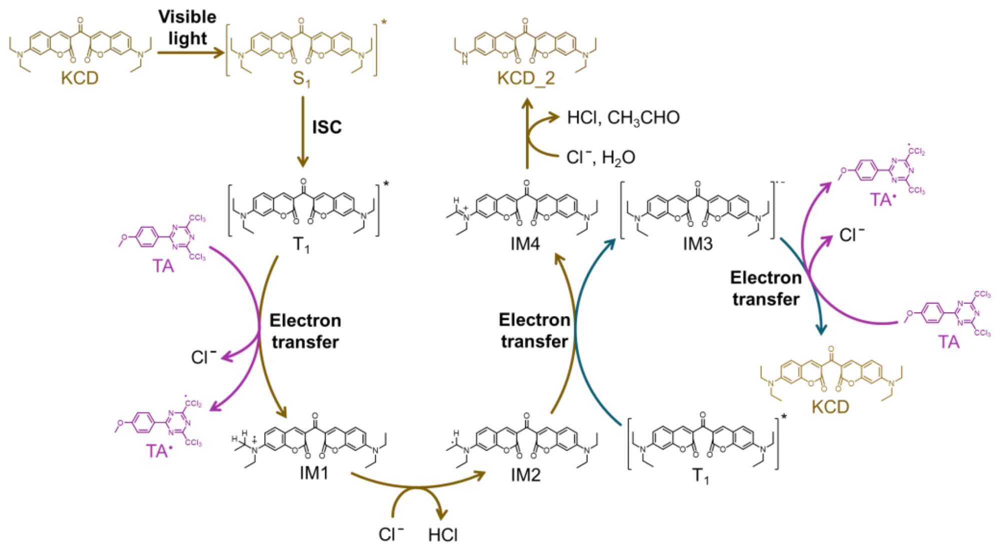

Supplementary Figure 3 | Proposed mechanism for the photooxidation of KCD by TA upon visible light irradiation.

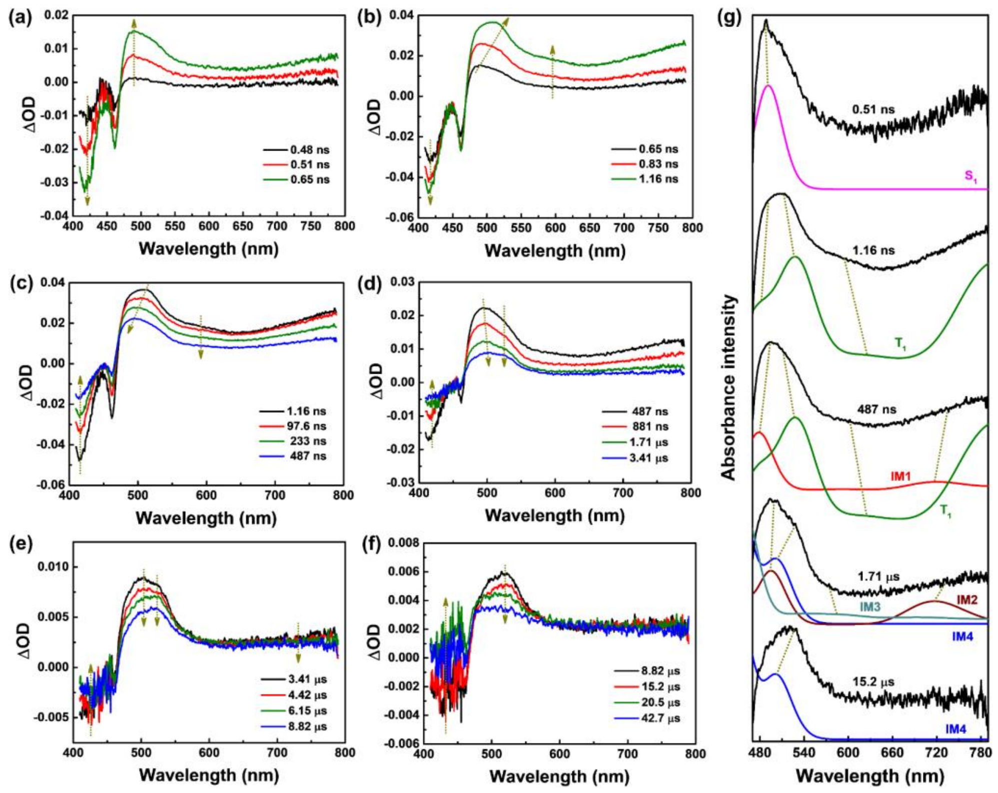

Supplementary Figure 4 | (a~f) Nanosecond transient absorption of KCD in the presence of TA upon exposure to 400 nm laser irradiation (power: 1.8 mW). The content of KCD and TA in toluene was 175.0 µM and 3.5 mM, respectively. (g) Experimental spectra (black lines) and the corresponding calculated ones by DFT. The calculated spectra were scaled by 1.23 times with a half-width of 1100 cm^{-1}.

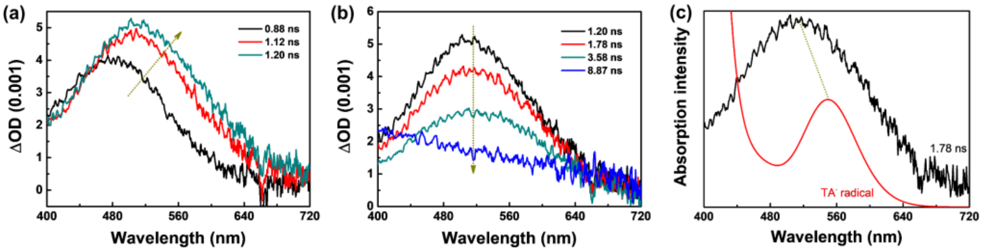

Supplementary Figure 5 | (a~b) Nanosecond transient absorption of pure TA in toluene upon 330 nm pulsed laser irradiation. (c) Experimental spectrum (black line) and the corresponding DFT calculated one (red line) of the radical generated by pure TA. The calculated spectrum was scaled by 1.30 times with a half-width of 1600 cm^{-1}. Results show that the radical $TA^{\cdot}$ can only last for 7.6 ns, showing a negligible influence on the transient absorption of the KCD/TA system.

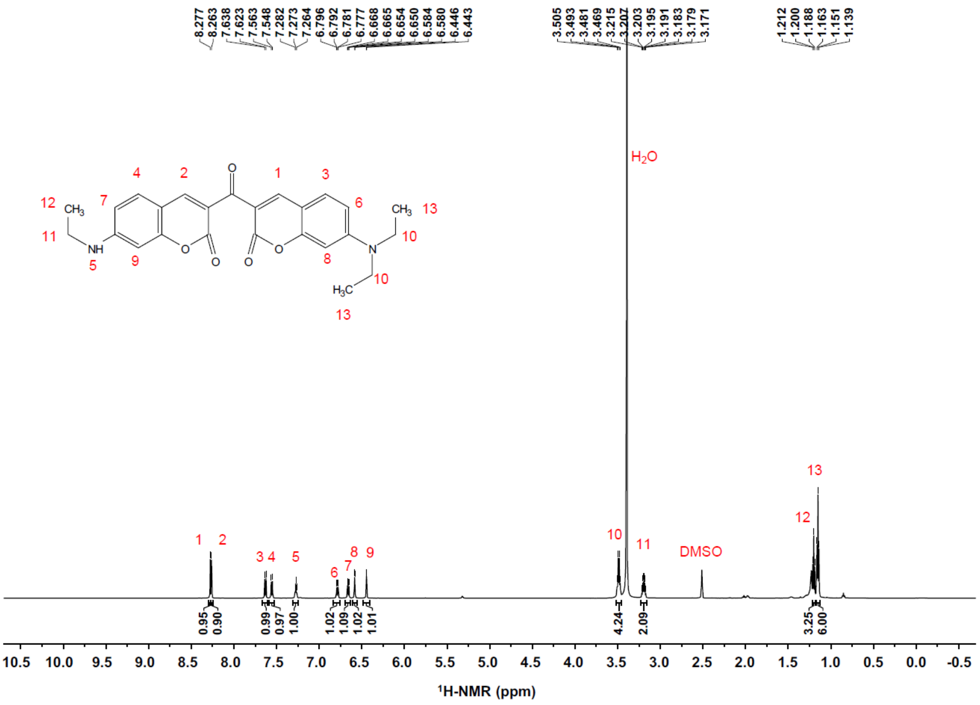

Supplementary Figure 6 | ^{1}H-NMR spectrum of KCD_2. ^{1}H-NMR (600 MHz, DMSO-d_6), δ (ppm): 8.28 (s, 1H), 8.26 (s, 1H), 7.64-7.62 (d, J = 9.0 Hz, 1H), 7.56-7.55 (d, J = 8.7 Hz, 1H), 7.28-7.26 (t, J = 5.2 Hz, 1H), 6.80-6.78 (dd, J_1 = 2.5 Hz, J_2 = 9.0 Hz, 1H), 6.67-6.65 (dd, J_1 = 2.1 Hz, J_2 = 8.7 Hz, 1H), 6.58 (d, J = 2.5 Hz, 1H), 6.45-6.44 (d, J = 2.1 Hz, 1H), 3.50-3.47 (q, J = 7.0 Hz, 4H), 3.21-3.17 (m, 2H), 1.21-1.19 (t, J = 7.2 Hz, 3H), 1.16-1.14 (t, J = 7.0 Hz, 6H).

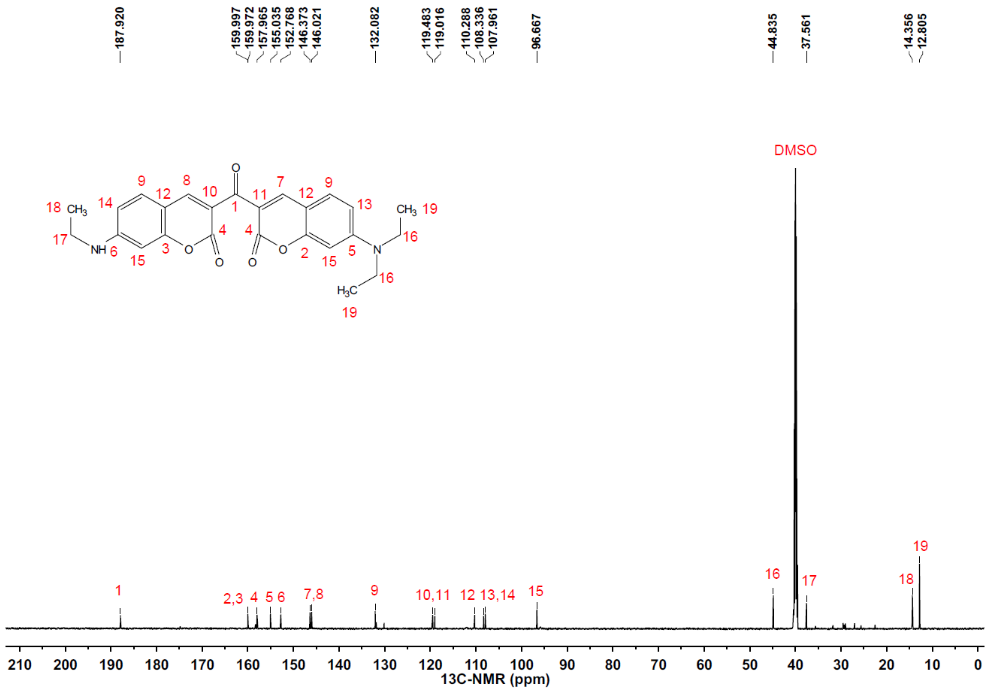

Supplementary Figure 7 | ^{13}C-NMR spectrum of KCD_2. ^{13}C-NMR (151 MHz, DMSO-d_6), δ (ppm): 187.92, 159.99, 159.97, 157.96, 155.03, 152.77, 146.37, 146.02, 132.08, 119.48, 119.02, 110.29, 108.34, 107.96, 96.67, 44.83, 37.56, 14.36, 12.80.

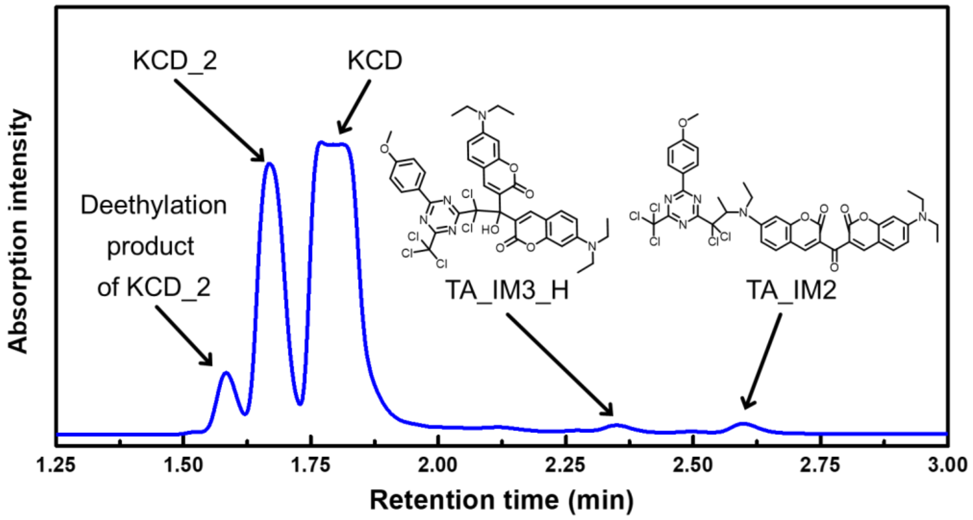

Supplementary Figure 8 | HPLC-HRMS spectrum monitored at the wavelength of 460 nm.

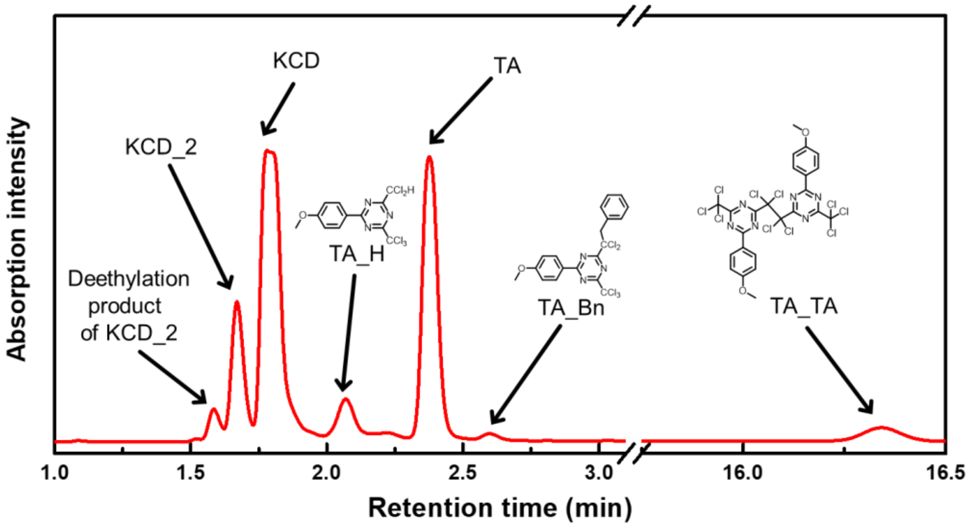

Supplementary Figure 9 | HPLC-HRMS spectrum monitored at the wavelength of 226 nm. Bn represents the benzyl functional group.

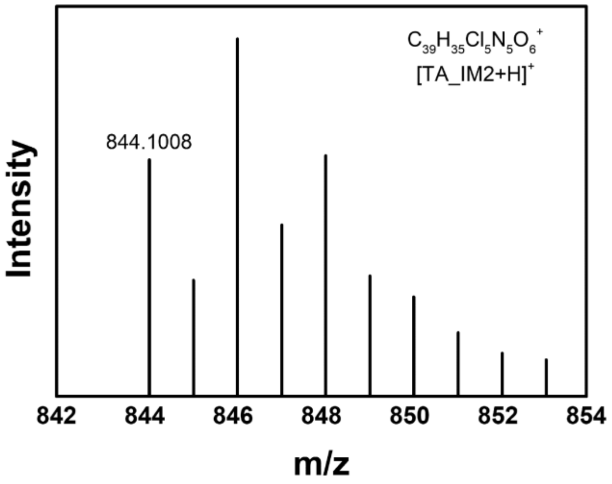

Supplementary Figure 10 | Found exact mass of [TA_IM2+H]$^+$ during the HPLC-HRMS characterization. The corresponding isotopic peaks are also presented.

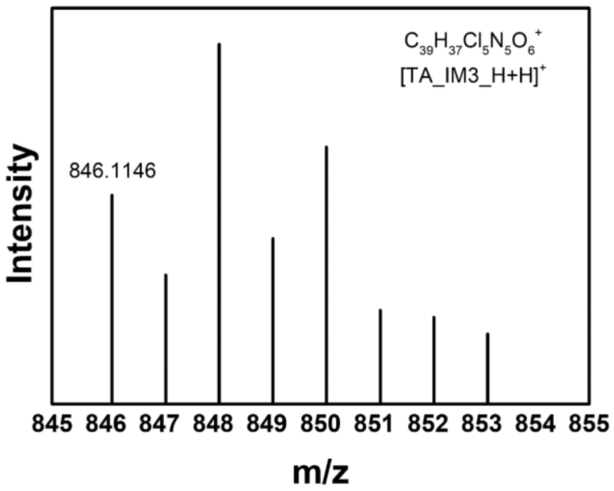

Supplementary Figure 11 | Found exact mass of [TA_IM3_H+H]$^+$ during the HPLC-HRMS characterization. The corresponding isotopic peaks are also presented.

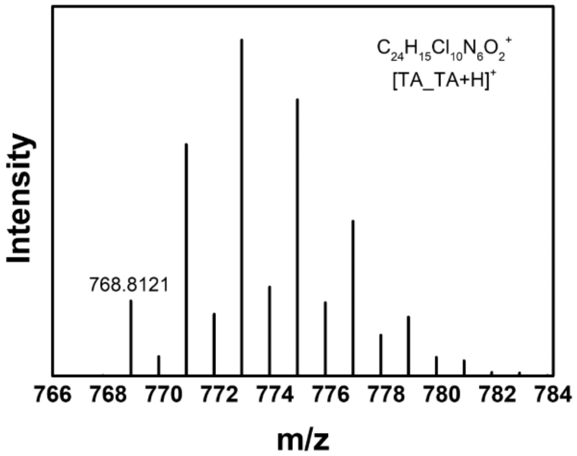

Supplementary Figure 12 | Found exact mass of [TA_TA+H]$^+$ during the HPLC-HRMS characterization. The corresponding isotopic peaks are also presented.

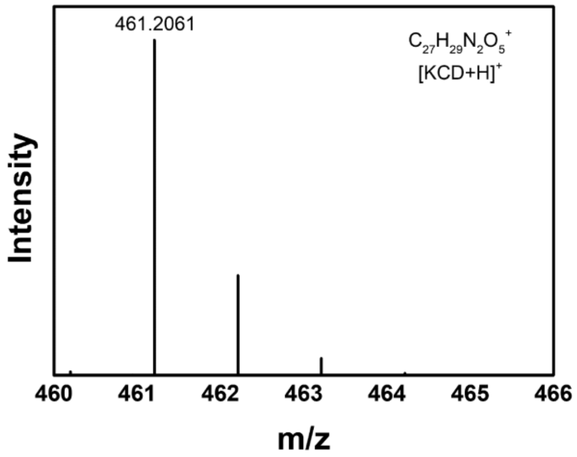

Supplementary Figure 13 | Found exact mass of [KCD+H]$^+$ during the HPLC-HRMS characterization. The corresponding isotopic peaks are also presented.

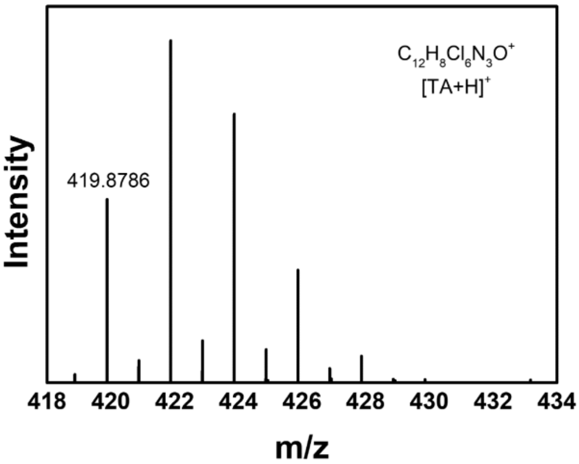

Supplementary Figure 14 | Found exact mass of $[TA+H]^+$ during the HPLC-HRMS characterization. The corresponding isotopic peaks are also presented.

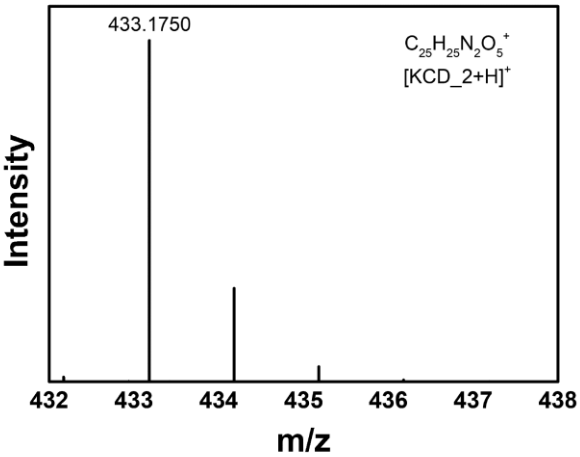

Supplementary Figure 15 | Found exact mass of [KCD_2+H]$^+$ during the HPLC-HRMS characterization. The corresponding isotopic peaks are also presented.

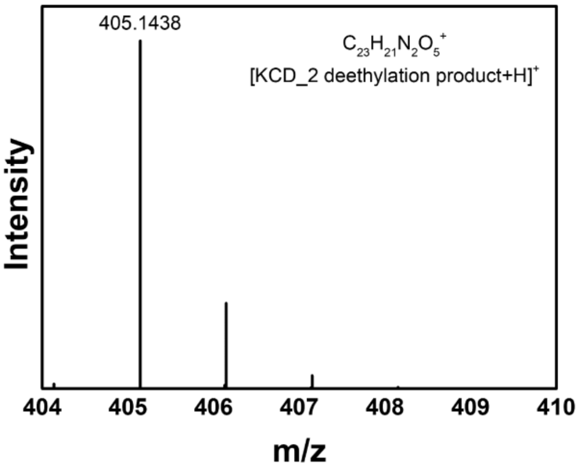

Supplementary Figure 16 | Found exact mass of [KCD_2 deethylation product+H]$^+$ during the HPLC-HRMS characterization. The corresponding isotopic peaks are also presented.

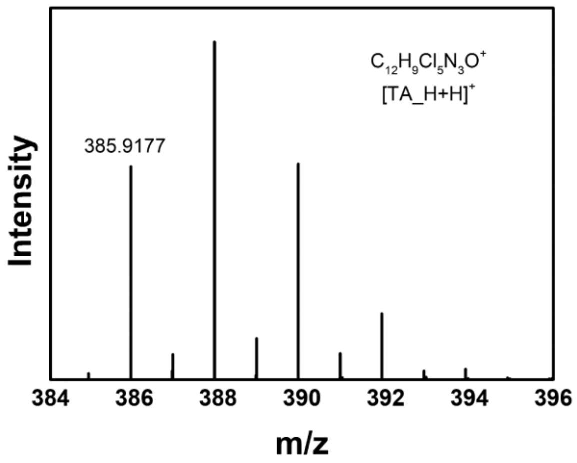

Supplementary Figure 17 | Found exact mass of [TA_H+H]$^+$ during the HPLC-HRMS characterization. The corresponding isotopic peaks are also presented.

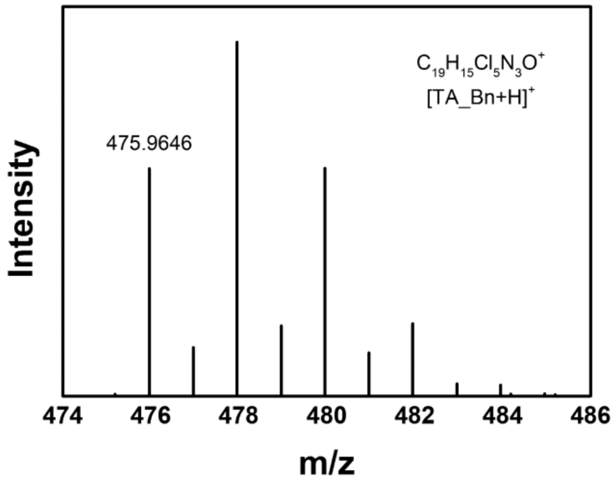

Supplementary Figure 18 | Found exact mass of [TA_Bn+H]$^+$ during the HPLC-HRMS characterization. The corresponding isotopic peaks are also presented. Bn represents the benzyl functional group.

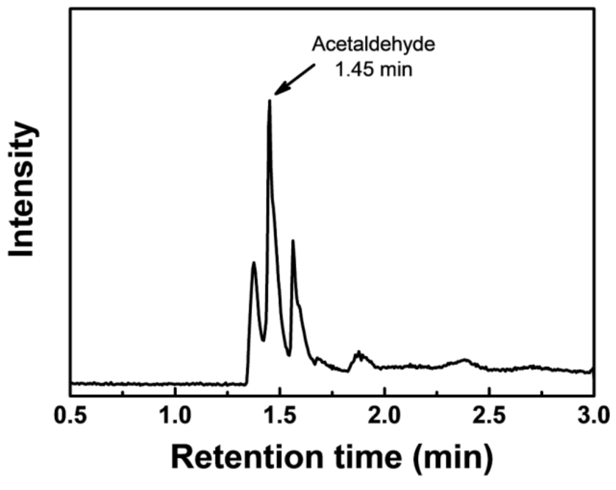

Supplementary Figure 19 | Spectrum obtained during the GC-MS characterization.

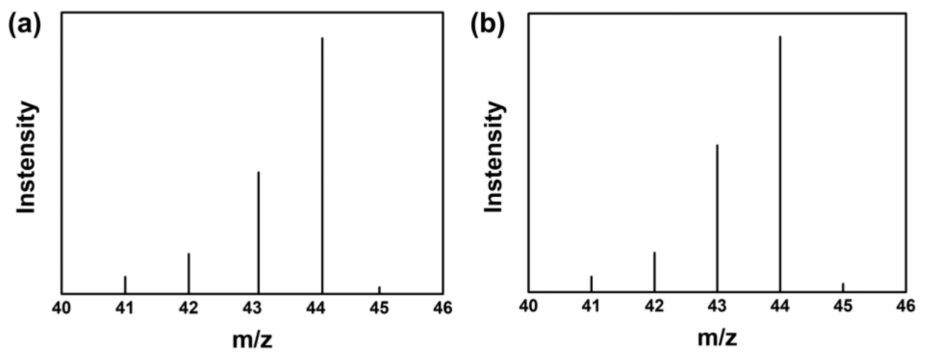

Supplementary Figure 20 | (a) Found mass spectrum at 1.45 min in GC-MS characterization and (b) the corresponding standard mass spectrum from the library provided by Bruker, confirming the generation of acetaldehyde.

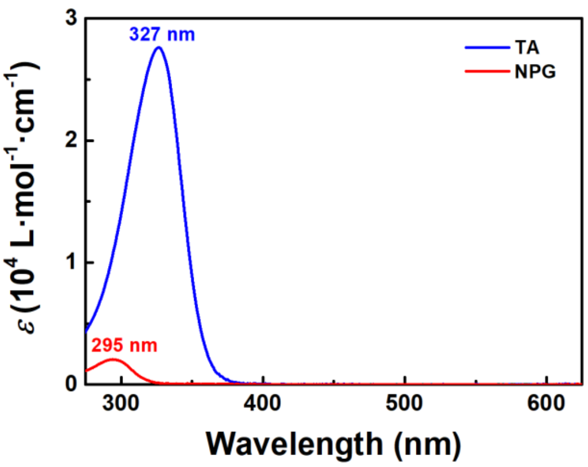

Supplementary Figure 21 | UV-vis absorption spectra of TA (blue line) and NPG (red line). NPG was recrystallized from hot water before use.

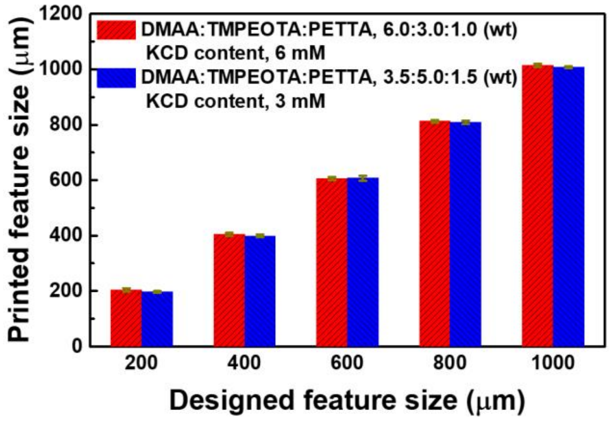

Supplementary Figure 22 | Printed feature size that was measured under an optical microscope (Axio Scope A1, Carl Zeiss) against that designed. The 3D printing was photomediated by the KCD/TA system with varied monomer weight ratio and KCD content. Averages with standard deviations (SD) are presented by measuring 6 square lattices for each entry.

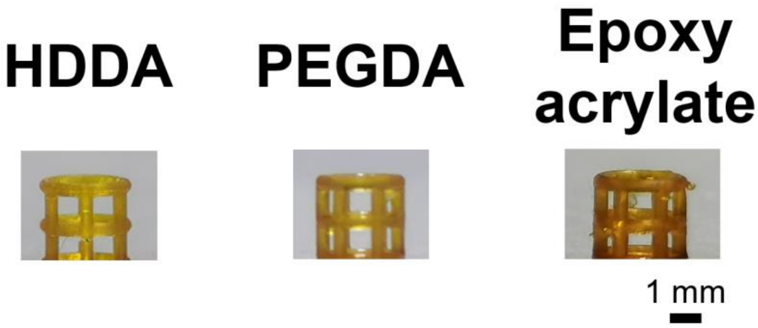

Supplementary Figure 23 | Images of 3D printed hollow cylinders using the monomer of HDDA, PEGDA and Epoxy acrylate.

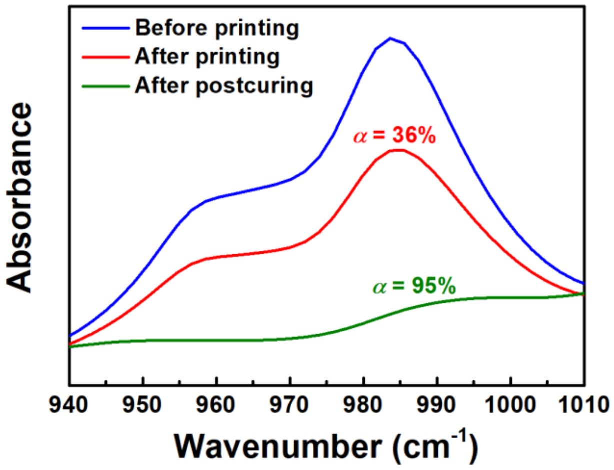

Supplementary Figure 24 | Characteristic peak absorptions of C=C bond before printing (blue line), after printing (red line) and after postcuring (green line), respectively.

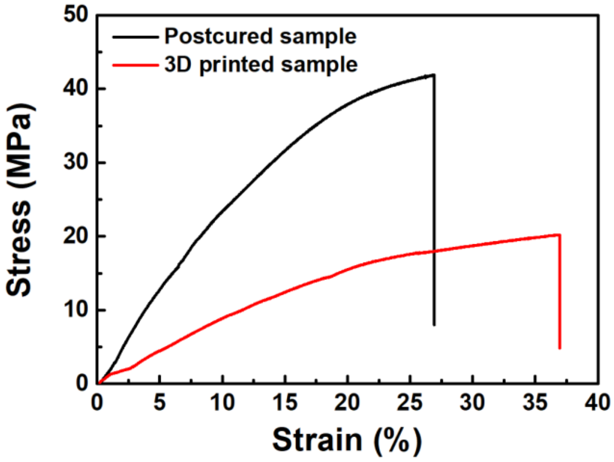

Supplementary Figure 25 | Stress against strain of 3D printed samples before (red line) and after (black line) postcuring. **NOTE: Complete experimental data are given in Supplementary Table 3.**

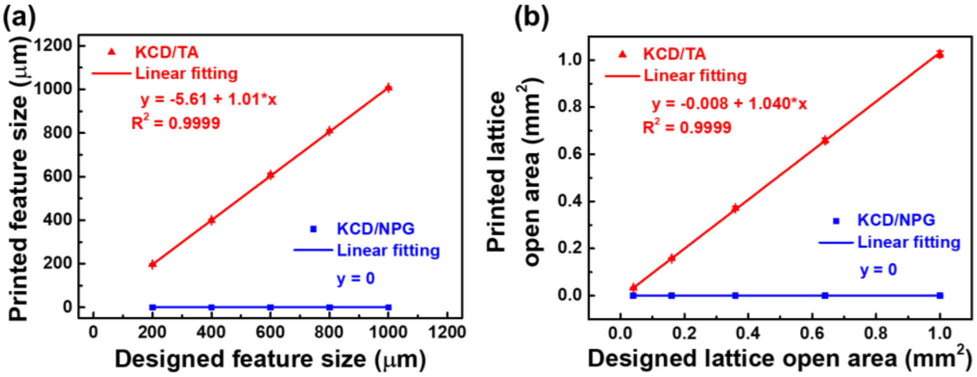

Supplementary Figure 26 | (a) Printed feature size and (b) lattice open area (measured under an optical microscope, Axio Scope A1, Carl Zeiss) photomediated by KCD/TA (red line) and KCD/NPG (blue line) against that designed. Averages with standard deviations (SD) are presented by measuring 6 square lattices for each entry. The printed feature sizes agree well with those predesigned with a deviation less than 1.6%.

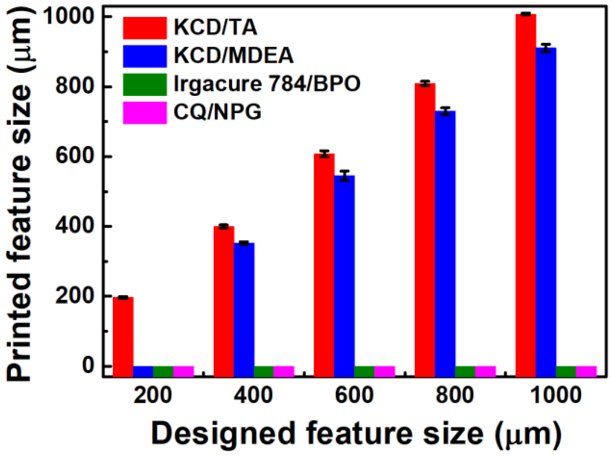

Supplementary Figure 27 | Printed feature size that was measured under an optical microscope (Axio Scope A1, Carl Zeiss) against that designed with different photosensitizers and co-initiators. Averages with standard deviations (SD) are presented by measuring 6 square lattices for each entry.

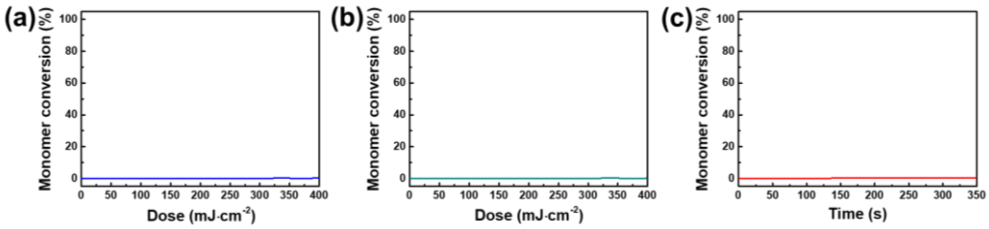

Supplementary Figure 28 | Monomer conversion mediated by (a) NPG and (b) TA that was measured by RT-FTIR (Vertex 80, Bruker) against irradiation dose of 460 nm light with light intensity of 1.2 mW·cm^{-2}; (c) Monomer conversion without light irradiation.

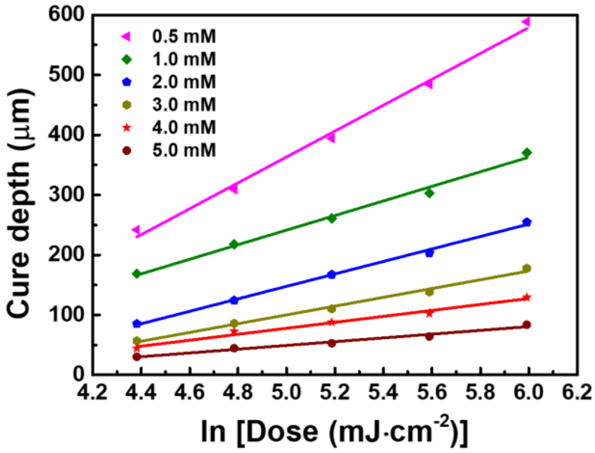

KCD (mM)	Slope	R^2
0.5	216	0.9921
1.0	121	0.9890
2.0	104	0.9962
3.0	73	0.9895
4.0	50	0.9785
5.0	31	0.9720

Supplementary Figure 29 | Cure depth of printing resins against irradiation dose at 460 nm when varying the KCD concentration: 0.5, 1.0, 2.0, 3.0, 4.0 and 5.0 mM, respectively. The TA concentration was 50 mM.

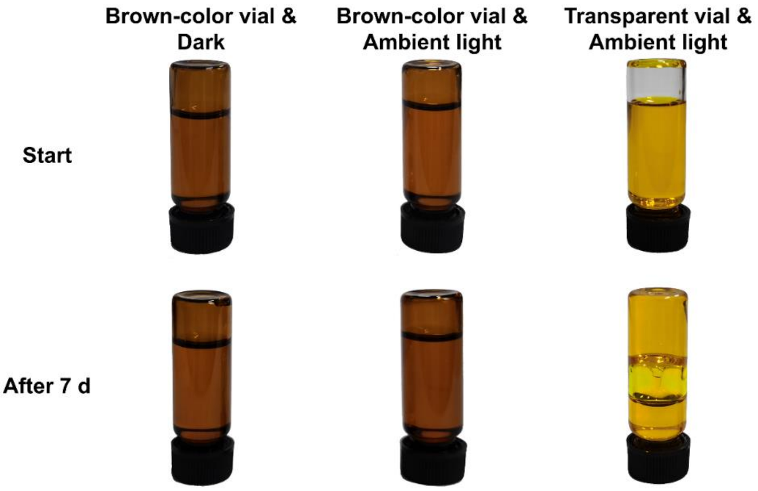

Supplementary Figure 30 | Appearance of the printing resin when kept in brown-color vial & dark condition, brown-color vial & ambient condition and transparent vial & ambient condition.

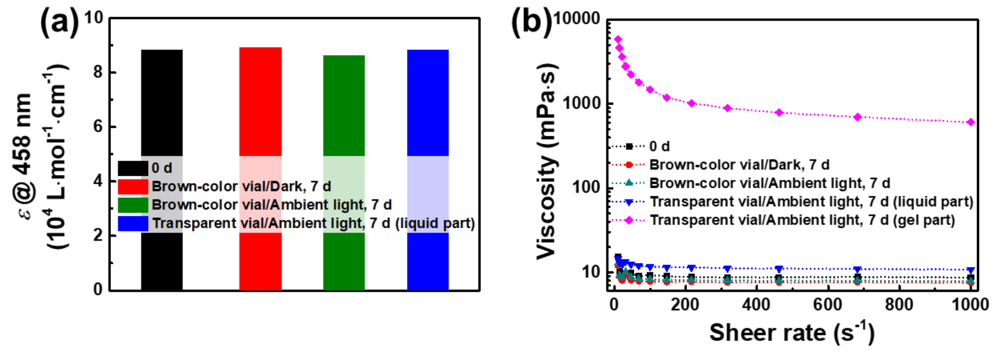

Supplementary Figure 31 | (a) Molar extinction coefficient (ε@ 458 nm) and (b) viscosity of printing resins when kept in brown-color vial & dark condition, brown-color vial & ambient condition and transparent vial & ambient condition.

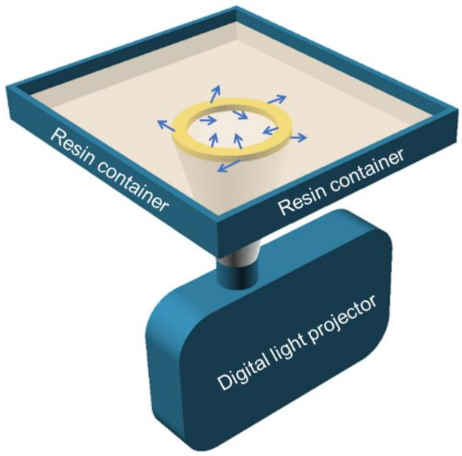

Supplementary Figure 32 | Illustration on the lateral photopolymerization of 3D printing.

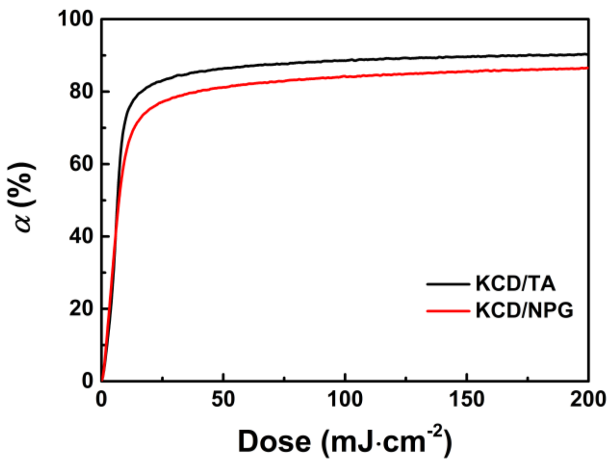

Supplementary Figure 33 | Double-bond conversion (α) measured by RT-FTIR (Vertex 80, Bruker) against irradiation dose. The photopolymerization was photomediated by the KCD/TA (black line) and KCD/NPG (red line) systems under 1.2 mW·cm^{-2} of 460 nm light, respectively. The contents of KCD, TA and NPG were 3.0, 50.0 and 50.0 mM, respectively.

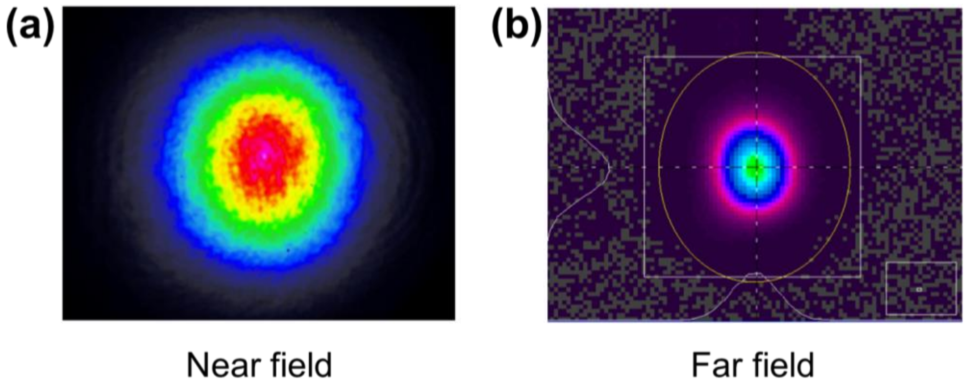

Supplementary Figure 34 | Laser beam spatial profile in (a) near field and (b) far field (focus of 1 m lens). Data was provided by the laser supplier and used with permission. Copyright @ Coherent.

4. Minimum Energy Geometry Optimized at the M06-2X/6-311G(d,p) Level

Supplementary Table 6 | Cartesian coordinates of the optimized M_KCD S_1

Center number	Atomic number	Cartesian coordinates/Angstroms		
		X	Y	Z
1	6	6.076815	0.869749	0.527311
2	6	6.062681	-0.466445	0.021066
3	6	4.828114	-1.005375	-0.413208
4	6	3.682681	-0.249605	-0.333521
5	6	3.677036	1.076491	0.170911
6	6	4.922860	1.603572	0.594425
7	8	2.538227	-0.819374	-0.785318
8	6	1.312861	-0.165211	-0.781900
9	6	1.264267	1.136469	-0.115859
10	6	2.446262	1.757577	0.250989
11	6	-0.000004	1.870923	-0.000051
12	8	0.000009	3.112155	0.000021
13	6	-1.264278	1.136478	0.115797
14	6	-2.446282	1.757590	-0.251000
15	6	-3.677054	1.076499	-0.170895
16	6	-3.682677	-0.249612	0.333494
17	8	-2.538206	-0.819388	0.785242
18	6	-1.312853	-0.165207	0.781831
19	6	-4.922893	1.603588	-0.594355
20	6	-6.076842	0.869756	-0.527227
21	6	-6.062686	-0.466454	-0.021028
22	6	-4.828103	-1.005392	0.413191
23	8	0.423009	-0.712980	-1.370055
24	8	-0.422951	-0.713027	1.369864
25	7	-7.203668	-1.199964	0.042760

26	6	-8.463731	-0.646072	-0.424478
27	6	-7.169262	-2.552063	0.574080
28	7	7.203669	-1.199946	-0.042715
29	6	8.463706	-0.646079	0.424626
30	6	7.169283	-2.552039	-0.574052
31	1	7.003561	1.311492	0.863700
32	1	4.742557	-2.006724	-0.808740
33	1	4.947893	2.614787	0.983788
34	1	2.396091	2.767335	0.640890
35	1	-2.396126	2.767354	-0.640887
36	1	-4.947943	2.614815	-0.983684
37	1	-7.003602	1.311507	-0.863571
38	1	-4.742530	-2.006752	0.808691
39	1	-8.405883	-0.369872	-1.481681
40	1	-8.175203	-2.962608	0.565408
41	1	8.405807	-0.369958	1.481846
42	1	6.800393	-2.559106	-1.604145
43	1	8.175230	-2.962567	-0.565386
44	1	-8.746003	0.239505	0.153316
45	1	-6.523897	-3.197182	-0.030518
46	1	9.242362	-1.394899	0.307964
47	1	-6.800369	-2.559142	1.604172
48	1	-9.242375	-1.394907	-0.307840
49	1	6.523925	-3.197177	0.030535
50	1	8.745997	0.239542	-0.153090

Supplementary Table 7 | Cartesian coordinates of the optimized M_KCD T_1

Center number	Atomic number	Cartesian coordinates/Angstroms X	Y	Z
1	6	-6.062304	0.855646	-0.468872
2	6	-6.017885	-0.495482	-0.017560
3	6	-4.771366	-1.023836	0.387762
4	6	-3.640831	-0.244403	0.333966
5	6	-3.663046	1.102033	-0.118826
6	6	-4.923944	1.615984	-0.513680
7	8	-2.487131	-0.808956	0.763203
8	6	-1.278870	-0.128768	0.785365
9	6	-1.251455	1.184908	0.127341
10	6	-2.453059	1.809016	-0.190132
11	6	-0.000006	1.927739	-0.000090
12	8	0.000002	3.168353	-0.000092
13	6	1.251448	1.184900	-0.127453
14	6	2.453040	1.809013	0.190055
15	6	3.663029	1.102029	0.118809
16	6	3.640834	-0.244413	-0.333969
17	8	2.487152	-0.808971	-0.763247
18	6	1.278886	-0.128792	-0.785448
19	6	4.923911	1.615987	0.513705
20	6	6.062275	0.855651	0.468951
21	6	6.017874	-0.495484	0.017656
22	6	4.771373	-1.023844	-0.387712
23	8	-0.382273	-0.647729	1.383395
24	8	0.382334	-0.647729	-1.383563
25	7	7.147321	-1.258895	-0.024018
26	6	8.419845	-0.710803	0.408413
27	6	7.075386	-2.632762	-0.486515

28	7	-7.147331	-1.258891	0.024173
29	6	-8.419856	-0.710834	-0.408301
30	6	-7.075369	-2.632768	0.486638
31	1	-6.999126	1.291829	-0.784568
32	1	-4.660221	-2.036590	0.746064
33	1	-4.971980	2.640058	-0.864958
34	1	-2.416948	2.832229	-0.544378
35	1	2.416917	2.832234	0.544278
36	1	4.971933	2.640067	0.864969
37	1	6.999084	1.291839	0.784677
38	1	4.660244	-2.036600	-0.746015
39	1	8.385299	-0.405369	1.459039
40	1	8.069333	-3.071911	-0.453970
41	1	-8.385306	-0.405457	-1.458944
42	1	-6.708468	-2.683836	1.516414
43	1	-8.069326	-3.071901	0.454192
44	1	8.705636	0.155367	-0.196398
45	1	6.412041	-3.230006	0.147158
46	1	-9.188741	-1.471528	-0.298432
47	1	6.708586	-2.683812	-1.516329
48	1	9.188734	-1.471499	0.298584
49	1	-6.412093	-3.230015	-0.147108
50	1	-8.705655	0.155366	0.196463

Supplementary Table 8 | Cartesian coordinates of the optimized M_IM1

Center number	Atomic number	Cartesian coordinates/Angstroms		
		X	Y	Z
1	6	6.084755	0.772840	0.030698
2	6	5.935429	-0.634226	-0.180643
3	6	4.629532	-1.162782	-0.291684
4	6	3.544972	-0.321470	-0.192310
5	6	3.679439	1.066081	0.017893
6	6	4.989256	1.582686	0.125966
7	8	2.313938	-0.888970	-0.313225
8	6	1.149388	-0.176811	-0.233452
9	6	1.273417	1.249881	0.024587
10	6	2.506457	1.835872	0.114171
11	6	0.065684	2.073160	0.111969
12	8	0.058235	3.281424	0.052760
13	6	-1.262984	1.368127	0.248099
14	6	-2.290213	1.672765	-0.588564
15	6	-3.504926	0.959343	-0.489564
16	6	-3.628906	-0.044079	0.518608
17	8	-2.645196	-0.258075	1.408448
18	6	-1.451329	0.445385	1.387132
19	6	-4.599365	1.157481	-1.371844
20	6	-5.732779	0.413605	-1.272305
21	6	-5.845427	-0.605083	-0.257886
22	6	-4.757560	-0.807205	0.636692
23	8	0.113805	-0.772487	-0.401686
24	8	-0.688914	0.300986	2.282299
25	7	-6.956472	-1.343389	-0.159444
26	6	-8.074709	-1.160167	-1.090569
27	6	-7.082163	-2.361322	0.887925

28	7	7.018668	-1.437469	-0.270611
29	6	8.360939	-0.884669	-0.150893
30	6	6.847425	-2.867905	-0.475430
31	1	7.069770	1.205785	0.120471
32	1	4.441477	-2.213678	-0.451642
33	1	5.116418	2.647049	0.288760
34	1	2.562285	2.909507	0.271580
35	1	-2.154397	2.426257	-1.357031
36	1	-4.512819	1.919829	-2.136903
37	1	-6.548749	0.591349	-1.956948
38	1	-4.781984	-1.550540	1.419699
39	1	-7.741701	-1.309470	-2.118564
40	1	-8.064044	-2.818128	0.819090
41	1	8.511940	-0.418278	0.826744
42	1	6.325535	-3.071514	-1.415019
43	1	7.825167	-3.339738	-0.518800
44	1	-8.502556	-0.161633	-0.982264
45	1	-6.321274	-3.133651	0.757815
46	1	9.083932	-1.688224	-0.260271
47	1	-6.973010	-1.904604	1.873196
48	1	-8.841235	-1.894456	-0.864942
49	1	6.282896	-3.318588	0.346186
50	1	8.554027	-0.143333	-0.931120

Supplementary Table 9 | Cartesian coordinates of the optimized M_IM2

Center number	Atomic number	Cartesian coordinates/Angstroms		
		X	Y	Z
1	6	-6.015404	0.818882	-0.586093
2	6	-6.000560	-0.486166	-0.021464
3	6	-4.779764	-0.991755	0.459315
4	6	-3.633499	-0.222661	0.369391
5	6	-3.635081	1.061333	-0.192355
6	6	-4.859909	1.557766	-0.665444
7	8	-2.493570	-0.767417	0.862070
8	6	-1.270471	-0.126667	0.830510
9	6	-1.243471	1.177605	0.162239
10	6	-2.389314	1.750948	-0.271600
11	6	0.039017	1.925266	0.033959
12	8	0.046814	3.137696	0.060994
13	6	1.316452	1.171645	-0.126759
14	6	2.462641	1.704767	0.352152
15	6	3.702582	1.006077	0.242885
16	6	3.690900	-0.244702	-0.391971
17	8	2.551935	-0.742594	-0.932796
18	6	1.336430	-0.087974	-0.877591
19	6	4.928872	1.470224	0.744329
20	6	6.079139	0.726268	0.628044
21	6	6.047212	-0.542469	-0.001674
22	6	4.832121	-1.014846	-0.520317
23	8	-0.351435	-0.661516	1.377863
24	8	0.419719	-0.571616	-1.472442
25	7	7.194805	-1.309118	-0.096532
26	6	8.487578	-0.696021	0.178556
27	6	7.162445	-2.644852	-0.409322

28	7	-7.143776	-1.230517	0.050860
29	6	-8.391804	-0.703578	-0.466125
30	6	-7.097678	-2.565278	0.615296
31	1	-6.936413	1.238424	-0.963343
32	1	-4.688713	-1.972893	0.901043
33	1	-4.885140	2.548935	-1.104895
34	1	-2.342444	2.744456	-0.707750
35	1	2.423154	2.670903	0.846382
36	1	4.957952	2.432925	1.242273
37	1	6.998918	1.107551	1.047896
38	1	4.760721	-1.947857	-1.059516
39	1	8.640559	-0.557897	1.253127
40	1	-8.321451	-0.478796	-1.535722
41	1	-6.759357	-2.545872	1.656460
42	1	-8.095274	-2.997436	0.589684
43	1	8.564773	0.269109	-0.323320
44	1	-9.173856	-1.446442	-0.327125
45	1	9.267499	-1.350621	-0.203565
46	1	-6.425581	-3.217014	0.046612
47	1	-8.691024	0.208485	0.060774
48	1	8.105712	-3.155455	-0.503133
49	1	6.244449	-3.192363	-0.277464

Supplementary Table 10 | Cartesian coordinates of the optimized M_IM3

Center	Atomic	Cartesian coordinates/Angstroms		
number	number	X	Y	Z
1	6	6.171629	0.886031	0.164619
2	6	6.108262	-0.501549	-0.050443
3	6	4.832402	-1.079203	-0.185796
4	6	3.692241	-0.305808	-0.086458
5	6	3.730597	1.086917	0.144715
6	6	5.018585	1.648448	0.262470
7	8	2.502427	-0.951181	-0.248798
8	6	1.264023	-0.308838	-0.190817
9	6	1.277629	1.102261	0.137129
10	6	2.506276	1.779695	0.239620
11	6	0.055569	1.856422	0.204356
12	8	0.034178	3.097877	0.165044
13	6	-1.266161	1.147754	0.274038
14	6	-2.312521	1.630777	-0.427028
15	6	-3.595442	0.980316	-0.411908
16	6	-3.730148	-0.171912	0.369169
17	8	-2.716121	-0.604121	1.152010
18	6	-1.486079	0.054402	1.237566
19	6	-4.710961	1.377737	-1.154791
20	6	-5.896856	0.666831	-1.132516
21	6	-6.012040	-0.509271	-0.360589
22	6	-4.907984	-0.906610	0.404834
23	8	0.318439	-1.008084	-0.460755
24	8	-0.763492	-0.286632	2.121382
25	7	-7.187258	-1.261669	-0.366891
26	6	-8.397985	-0.621335	-0.837523

27	6	-7.345521	-2.268607	0.662360
28	7	7.274818	-1.298617	-0.104412
29	6	8.510396	-0.602419	-0.396157
30	6	7.142829	-2.578055	-0.773146
31	1	7.126482	1.384477	0.262232
32	1	4.686905	-2.133877	-0.371010
33	1	5.099624	2.716945	0.432336
34	1	2.483070	2.847902	0.414514
35	1	-2.158268	2.512965	-1.040185
36	1	-4.636074	2.272140	-1.764452
37	1	-6.728964	1.020657	-1.723804
38	1	-4.920668	-1.779933	1.040296
39	1	-8.311513	-0.360145	-1.894405
40	1	-8.309536	-2.759181	0.531852
41	1	8.756680	0.093799	0.407657
42	1	6.778004	-2.484899	-1.808738
43	1	8.114505	-3.073238	-0.784268
44	1	-8.643053	0.292941	-0.276791
45	1	-6.571369	-3.032881	0.569723
46	1	9.320822	-1.329784	-0.455873
47	1	-7.294216	-1.850676	1.678734
48	1	-9.226839	-1.322058	-0.742995
49	1	6.453799	-3.225079	-0.228553
50	1	8.471811	-0.034851	-1.340436

Supplementary Table 11 | Cartesian coordinates of the optimized M_IM4

Center number	Atomic number	Cartesian coordinates/Angstroms X	Y	Z
1	6	6.058064	0.836475	0.091984
2	6	5.950425	-0.570325	-0.131188
3	6	4.662995	-1.131381	-0.268255
4	6	3.552873	-0.318375	-0.185954
5	6	3.646710	1.067862	0.034022
6	6	4.937938	1.617346	0.170914
7	8	2.341570	-0.915867	-0.335977
8	6	1.149680	-0.237083	-0.281685
9	6	1.231895	1.194527	-0.009997
10	6	2.446181	1.805786	0.108554
11	6	0.008882	2.014640	0.052180
12	8	0.021054	3.219100	-0.057730
13	6	-1.318829	1.315997	0.213242
14	6	-2.350061	1.647642	-0.575511
15	6	-3.598631	0.926673	-0.476276
16	6	-3.675807	-0.102220	0.473971
17	8	-2.673339	-0.367584	1.324080
18	6	-1.468501	0.337882	1.305972
19	6	-4.703787	1.173020	-1.295907
20	6	-5.862212	0.423759	-1.186798
21	6	-5.891182	-0.604529	-0.246170
22	6	-4.823433	-0.877652	0.595059
23	8	0.144341	-0.865910	-0.485052
24	8	-0.692461	0.134043	2.177121
25	7	-7.074526	-1.432457	-0.129524
26	6	-8.378741	-0.741234	-0.066176
27	6	-6.984977	-2.703405	-0.047774

28	7	7.059313	-1.345736	-0.209679
29	6	8.382293	-0.756785	-0.069202
30	6	6.928210	-2.775954	-0.433371
31	1	7.029161	1.295330	0.203638
32	1	4.504329	-2.185533	-0.438453
33	1	5.034109	2.683636	0.342496
34	1	2.471313	2.879614	0.271052
35	1	-2.232397	2.426369	-1.321595
36	1	-4.642048	1.962612	-2.035213
37	1	-6.696768	0.613523	-1.847985
38	1	-4.862836	-1.637486	1.366373
39	1	-8.555618	-0.243350	-1.017780
40	1	8.509894	-0.293587	0.913662
41	1	6.425803	-2.982813	-1.383265
42	1	7.918283	-3.222764	-0.466751
43	1	-8.329384	-0.002357	0.731980
44	1	9.129414	-1.538679	-0.176321
45	1	-9.161883	-1.471237	0.121796
46	1	6.362488	-3.252173	0.373291
47	1	8.564988	-0.002967	-0.840459
48	1	-7.880685	-3.295155	0.102447
49	1	-6.010912	-3.171884	-0.133834

Supplementary Table 12 | Cartesian coordinates of the optimized TA$^{\bullet}$

**Center number**	**Atomic number**	**Cartesian coordinates/Angstroms**		
		**X**	**Y**	**Z**
1	6	2.120735	-0.125845	-0.020872
2	6	3.003684	0.951954	0.006174
3	6	4.376640	0.748212	0.013314
4	6	4.877039	-0.556253	-0.007792
5	6	3.994443	-1.643931	-0.035561
6	6	2.633275	-1.430876	-0.042104
7	6	0.669380	0.100208	-0.029915
8	6	-1.106736	1.491489	-0.028584
9	6	-1.425728	-0.710161	-0.058978
10	1	2.603184	1.957816	0.021986
11	1	5.039366	1.601936	0.034757
12	1	4.413945	-2.641614	-0.052276
13	1	1.944099	-2.265593	-0.064418
14	6	-1.646533	2.827466	-0.014688
15	6	-2.368050	-1.925331	-0.010694
16	8	6.188642	-0.869887	-0.003952
17	6	7.128519	0.187182	0.024454
18	1	8.107899	-0.284694	0.022029
19	1	7.029830	0.827014	-0.857666
20	1	7.016280	0.789928	0.930743
21	17	-3.317034	3.098151	-0.020165
22	17	-3.971007	-1.526118	-0.645031
23	17	-2.504155	-2.387396	1.710644
24	17	-1.691517	-3.279025	-0.934665
25	7	-1.989740	0.478208	-0.044999
26	7	0.220037	1.357337	-0.025858
27	7	-0.132735	-0.976754	-0.043151

28	17	-0.627269	4.181334	0.008394

5. Minimum Energy Geometry Optimized at the B3LYP/6-31+G(d) Level

Supplementary Table 13 | Cartesian coordinates of the optimized M_IM2

Center number	Atomic number	Cartesian coordinates/Angstroms		
		X	Y	Z
1	6	6.042639	0.829667	0.645331
2	6	6.063596	-0.462649	0.036779
3	6	4.851323	-0.976339	-0.478747
4	6	3.685572	-0.228782	-0.382200
5	6	3.651378	1.046510	0.222434
6	6	4.868271	1.550298	0.730900
7	8	2.553664	-0.775227	-0.911069
8	6	1.306010	-0.147053	-0.871523
9	6	1.248652	1.136738	-0.173384
10	6	2.395934	1.711669	0.300176
11	6	-0.040579	1.873134	-0.042489
12	8	-0.049531	3.104888	-0.072066
13	6	-1.321761	1.128201	0.124448
14	6	-2.475646	1.666371	-0.377093
15	6	-3.723571	0.991910	-0.265164
16	6	-3.741773	-0.255440	0.399523
17	8	-2.605856	-0.758883	0.959760
18	6	-1.365351	-0.116509	0.891679
19	6	-4.948495	1.466026	-0.785404
20	6	-6.116360	0.739489	-0.658343
21	6	-6.113535	-0.521306	0.001817
22	6	-4.900728	-1.005507	0.537535
23	8	0.399283	-0.697543	-1.457325

24	8	-0.455782	-0.621664	1.511418
25	7	-7.283934	-1.268274	0.109427
26	6	-8.571523	-0.631927	-0.179260
27	6	-7.291943	-2.599646	0.439267
28	7	7.229656	-1.186278	-0.045420
29	6	8.469669	-0.652842	0.500917
30	6	7.225565	-2.506582	-0.660085
31	1	6.951038	1.257682	1.050376
32	1	4.791859	-1.947000	-0.953502
33	1	4.873260	2.529859	1.203087
34	1	2.333221	2.691874	0.767445
35	1	-2.424079	2.623547	-0.890840
36	1	-4.963057	2.420581	-1.305511
37	1	-7.027348	1.132175	-1.093322
38	1	-4.848784	-1.931383	1.095958
39	1	-8.721292	-0.509549	-1.259331
40	1	8.392070	-0.467865	1.581328
41	1	6.902545	-2.461416	-1.709074
42	1	8.236240	-2.916437	-0.635066
43	1	-8.633128	0.345253	0.307148
44	1	9.269880	-1.376652	0.340019
45	1	-9.368869	-1.265612	0.211423
46	1	6.562139	-3.200164	-0.125007
47	1	8.758967	0.286096	0.009244
48	1	-8.247106	-3.085992	0.567942
49	1	-6.375889	-3.168281	0.401187

Supplementary Table 14| Cartesian coordinates of the optimized M_IM2 - e^-

Center	Atomic	Cartesian coordinates/Angstroms		
number	number	X	Y	Z
1	6	6.104758	0.855190	0.092342
2	6	6.014234	-0.557528	-0.139946
3	6	4.724125	-1.130616	-0.285190
4	6	3.602510	-0.327028	-0.200940
5	6	3.678092	1.070043	0.029120
6	6	4.972933	1.630683	0.172942
7	8	2.388449	-0.936705	-0.355150
8	6	1.181043	-0.258316	-0.288355
9	6	1.247040	1.170993	-0.016975
10	6	2.472551	1.794548	0.109227
11	6	0.016408	1.970323	0.069000
12	8	0.012881	3.197049	0.007414
13	6	-1.315871	1.266759	0.211535
14	6	-2.336296	1.563571	-0.629002
15	6	-3.596984	0.876425	-0.523325
16	6	-3.733875	-0.090002	0.498161
17	8	-2.751956	-0.314040	1.397728
18	6	-1.514660	0.358496	1.353402
19	6	-4.687145	1.094430	-1.386317
20	6	-5.870288	0.379943	-1.252647
21	6	-5.959853	-0.593151	-0.243140
22	6	-4.905600	-0.830271	0.643380
23	8	0.171471	-0.911860	-0.486520
24	8	-0.756395	0.176804	2.266203
25	7	-7.162178	-1.382285	-0.112727
26	6	-8.465558	-0.694804	-0.301760
27	6	-7.123985	-2.637718	0.186073

28	7	7.137215	-1.327009	-0.218948
29	6	8.462931	-0.730202	-0.064901
30	6	7.027672	-2.763984	-0.457967
31	1	7.072456	1.326078	0.208049
32	1	4.581465	-2.188536	-0.461235
33	1	5.063528	2.699065	0.351392
34	1	2.490921	2.867739	0.285460
35	1	-2.191900	2.297918	-1.417100
36	1	-4.594171	1.832092	-2.178015
37	1	-6.682307	0.554110	-1.948995
38	1	-4.982971	-1.527006	1.471782
39	1	-8.571510	-0.413362	-1.350971
40	1	8.580265	-0.262168	0.920478
41	1	6.531758	-2.972346	-1.414612
42	1	8.027026	-3.197665	-0.492166
43	1	-8.481673	0.198579	0.324356
44	1	9.216308	-1.512154	-0.159263
45	1	-9.271540	-1.374059	-0.025094
46	1	6.466261	-3.260543	0.343807
47	1	8.656455	0.023277	-0.838689
48	1	-8.048746	-3.179405	0.353433
49	1	-6.167579	-3.143249	0.263403

Supplementary Table 15 | Cartesian coordinates of the optimized M_IM3

**Center**	**Atomic**	**Cartesian coordinates/Angstroms**		
**number**	**number**	**X**	**Y**	**Z**
1	6	-6.156786	0.946580	-0.457027
2	6	-6.199546	-0.367819	0.070900
3	6	-4.979616	-0.945933	0.483969
4	6	-3.790438	-0.230458	0.387616
5	6	-3.729257	1.081709	-0.132520

6	6	-4.957221	1.643516	-0.547271
7	8	-2.657191	-0.847749	0.831996
8	6	-1.377252	-0.248555	0.791431
9	6	-1.282082	1.046607	0.122593
10	6	-2.452296	1.700277	-0.240027
11	6	0.000003	1.759699	-0.000002
12	8	0.000002	3.021793	-0.000023
13	6	1.282088	1.046604	-0.122565
14	6	2.452303	1.700292	0.240027
15	6	3.729265	1.081728	0.132526
16	6	3.790450	-0.230449	-0.387584
17	8	2.657202	-0.847756	-0.831941
18	6	1.377256	-0.248583	-0.791355
19	6	4.957232	1.643553	0.547250
20	6	6.156801	0.946624	0.457005
21	6	6.199562	-0.367788	-0.070898
22	6	4.979632	-0.945916	-0.483940
23	8	-0.508784	-0.858690	1.380523
24	8	0.508788	-0.858730	-1.380435
25	7	7.419981	-1.063948	-0.211988
26	6	8.548614	-0.617317	0.588452
27	6	7.355572	-2.500371	-0.431363
28	7	-7.419997	-1.063935	0.211956
29	6	-8.548547	-0.617222	-0.588552
30	6	-7.355708	-2.500369	0.431314
31	1	-7.062337	1.434243	-0.799174
32	1	-4.920881	-1.949496	0.886135
33	1	-4.955979	2.649278	-0.962747
34	1	-2.367179	2.704755	-0.644997

35	1	2.367185	2.704783	0.644964
36	1	4.955987	2.649325	0.962701
37	1	7.062346	1.434308	0.799129
38	1	4.920910	-1.949488	-0.886091
39	1	8.355600	-0.667751	1.676350
40	1	8.372041	-2.886613	-0.550999
41	1	-8.355495	-0.667740	-1.676440
42	1	-6.808859	-2.726449	1.351842
43	1	-8.372217	-2.886531	0.550876
44	1	8.819050	0.413919	0.339731
45	1	6.868353	-3.044619	0.398607
46	1	-9.415304	-1.244488	-0.360846
47	1	6.808635	-2.726387	-1.351858
48	1	9.415289	-1.244698	0.360748
49	1	-6.868480	-3.044643	-0.398633
50	1	-8.818862	0.414063	-0.339899

Supplementary Table 16 | Cartesian coordinates of the optimized M_IM3 - e^-

Center number	Atomic number	Cartesian coordinates/Angstroms		
		X	Y	Z
1	6	-6.076829	0.796187	-0.674340
2	6	-6.093327	-0.480185	-0.033176
3	6	-4.880253	-0.974700	0.498762
4	6	-3.717110	-0.225425	0.384088
5	6	-3.686937	1.033136	-0.254530
6	6	-4.904899	1.518687	-0.777581
7	8	-2.584315	-0.752195	0.930644
8	6	-1.339426	-0.118647	0.879239
9	6	-1.285828	1.145890	0.146420

10	6	-2.434063	1.701837	-0.346410
11	6	-0.000001	1.886566	-0.000012
12	8	0.000000	3.118523	-0.000011
13	6	1.285828	1.145890	-0.146437
14	6	2.434060	1.701839	0.346397
15	6	3.686935	1.033138	0.254524
16	6	3.717112	-0.225424	-0.384091
17	8	2.584319	-0.752196	-0.930651
18	6	1.339429	-0.118649	-0.879252
19	6	4.904894	1.518691	0.777579
20	6	6.076826	0.796191	0.674345
21	6	6.093327	-0.480183	0.033184
22	6	4.880255	-0.974699	-0.498758
23	8	-0.432915	-0.648125	1.484180
24	8	0.432921	-0.648128	-1.484197
25	7	7.256250	-1.207520	-0.063434
26	6	8.503993	-0.678703	0.469337
27	6	7.251245	-2.504297	-0.726030
28	7	-7.256250	-1.207522	0.063448
29	6	-8.503997	-0.678703	-0.469313
30	6	-7.251244	-2.504293	0.726056
31	1	-6.986391	1.210217	-1.091143
32	1	-4.818378	-1.930845	1.001831
33	1	-4.912998	2.485588	-1.275141
34	1	-2.374523	2.669426	-0.839670
35	1	2.374518	2.669429	0.839653
36	1	4.912992	2.485593	1.275136
37	1	6.986386	1.210222	1.091151
38	1	4.818383	-1.930846	-1.001824

39	1	8.439086	-0.498735	1.551138
40	1	8.255830	-2.928202	-0.691130
41	1	-8.439095	-0.498730	-1.551112
42	1	-6.952445	-2.420018	1.780316
43	1	-8.255830	-2.928198	0.691162
44	1	8.790310	0.262170	-0.021129
45	1	6.567991	-3.206778	-0.229803
46	1	-9.300365	-1.404130	-0.297023
47	1	6.952447	-2.420031	-1.780290
48	1	9.300363	-1.404128	0.297048
49	1	-6.567992	-3.206779	0.229835
50	1	-8.790311	0.262168	0.021159

6. Supplementary References

1. Walker, D. A., Hedrick, J. L. & Mirkin, C. A. Rapid, large-volume, thermally controlled 3D printing using a mobile liquid interface. *Science* **366**, 360-364 (2019).
2. Sutton, J. T., Rajan, K., Harper, D. P. & Chmely, S. C. Lignin-containing photoactive resins for 3D printing by stereolithography. *ACS Appl. Mater. Interfaces* **10**, 36456-36463 (2018).
3. Majima, T. & Schnabel, W. On the reactivity of phosphinoyl and thiophosphinoyl radicals: Flash photolysis studies. *J. Photochem. Photobiol., A* **50**, 31-39 (1989).
4. Dietlin, C. *et al.* Rational design of acyldiphenylphosphine oxides as photoinitiators of radical polymerization. *Macromolecules* **52**, 7886-7893 (2019).
5. Wang, J. *et al.* A highly efficient waterborne photoinitiator for visible-light-induced three-dimensional printing of hydrogels. *Chem. Commun.* **54**, 920-923 (2018).
6. Wamser, C. C. I. Photochemical studies of phenoxy radical II. The photoreaction of Michler's ketone with benzophenone. Califormia Institute of Technology (1970).
7. Williams, J. L. R., Specht, D. P. & Farid, S. Ketocoumarins as photosensitizers and photoinitiators. *Polym. Eng. Sci.* **23**, 1022-1024 (1983).
8. Peng, H. Y. *et al.* Classical photopolymerization kinetics, exceptional gelation, and improved diffraction efficiency and driving voltage in scaffolding morphological H-PDLCs afforded using a photoinitibitor. *Polym. Chem.* **6**, 8259-8269 (2015).
9. Frisch, M. J. *et al.* Gaussion. In: *Gaussion 09* (ed D01). D.01 edn. Gaussion, Inc. (2013).
10. Fu, Y., Liu, L., Yu, H. Z., Wang, Y. M. & Guo, Q. X. Quantum-chemical predictions of absolute standard redox potentials of diverse organic molecules and free radicals in acetonitrile. *J. Am. Chem. Soc.* **127**, 7227-7234 (2005).

11. Marenich, A. V., Cramer, C. J. & Truhlar, D. G. Universal solvation model based on solute electron density and on a continuum model of the solvent defined by the bulk dielectric constant and atomic surface tensions. *J. Phys. Chem. B* **113**, 6378-6396 (2009).

12. Trasatti, S. The absolute electrode potential: An explanatory note. *Pure Appl. Chem.* **58**, 955-966 (1986).

13. Grotzinger, C., Burget, D., Jacques, P. & Fouassier, J. P. Photopolymerization reactions initiated by a visible light photoinitiating system: Dye/amine/bis(trichloromethyl)-substituted-1,3,5-triazine. *Macromol. Chem. Phys.* **202**, 3513-3522 (2001).

14. Pohlers, G., Scaiano, J. C., Step, E. & Sinta, R. Ionic vs free radical pathways in the direct and sensitized photochemistry of 2-(4'-methoxynaphthyl)-4,6-bis(trichloromethyl)-1,3,5-triazine: Relevance for photoacid generation. *J. Am. Chem. Soc.* **121**, 6167-6175 (1999).

15. Maroz, A., Hermann, R., Naumov, S. & Brede, O. Ionization of aniline and its *N*-methyl and *N*-phenyl substituted derivatives by (free) electron transfer to *N*-butyl chloride parent radical cations. *J. Phys. Chem. A* **109**, 4690-4696 (2005).

16. Peng, H. Y. *et al.* Monochromatic visible light "photoinitibitor": Janus-faced initiation and inhibition for storage of colored 3D images. *J. Am. Chem. Soc.* **136**, 8855-8858 (2014).

17. Specht, D. P., Martic, P. A. & Farid, S. Ketocoumarins: A new class of triplet sensitizers. *Tetrahedron* **38**, 1203-1211 (1982).

18. Zhao, X. Y. *et al.* Effect of ketyl radical on the structure and performance of holographic polymer/liquid-crystal composites. *Sci. China Mater.* **62**, 1921-1933 (2019).

19. Tumbleston, J. R. *et al.* Continuous liquid interface production of 3D objects. *Science* **347**, 1349-1352 (2015).

20. Zheng, X. *et al.* Ultralight, ultrastiff mechanical metamaterials. *Science* **344**, 1373-1377 (2014).

21. Han, D. *et al.* 4D printing of a bioinspired microneedle array with backward-facing barbs for enhanced tissue adhesion. *Adv. Funct. Mater.* **30**, 1909197 (2020).

22. Gong, H., Bickham, B. P., Woolley, A. T. & Nordin, G. P. Custom 3D printer and resin for 18 μm × 20 μm microfluidic flow channels. *Lab Chip* **17**, 2899-2909 (2017).

23. Metral, B., Bischoff, A., Ley, C., Ibrahim, A. & Allonas, X. Photochemical study of a three-component photocyclic initiating system for free radical photopolymerization: Implementing a model for digital light processing 3D printing. *ChemPhotoChem* **3**, 1109-1118 (2019).

24. Nejadebrahim, A. *et al.* A new safranin based three-component photoinitiating system for high resolution and low shrinkage printed parts via digital light processing. *RSC Adv.* **9**, 39709-39720 (2019).

25. Bagheri, A. *et al.* 3D printing of polymeric materials based on photo-RAFT polymerization. *Polym. Chem.* **11**, 641-647 (2020).

26. Bagheri, A. *et al.* Oxygen tolerant PET-RAFT facilitated 3D printing of polymeric materials under visible LEDs. *ACS Appl. Polym. Mater.* **2**, 782-790 (2020).

27. Zhang, Z., Corrigan, N., Bagheri, A., Jin, J. & Boyer, C. A versatile 3D and 4D printing system through photocontrolled RAFT polymerization. *Angew. Chem. Int. Ed.* **58**, 17954-17963 (2019).

28. Lee, K., Corrigan, N. & Boyer, C. Rapid high-resolution 3D printing and surface functionalization via type I photoinitiated RAFT polymerization. *Angew. Chem. Int. Ed.* **133**, 8839-8850 (2021).
